# Fertility clinics have a duty of care towards patients who do not have children with treatment

**DOI:** 10.1093/humrep/deae128

**Published:** 2024-06-18

**Authors:** S Gameiro, D Leone, H Mertes

**Affiliations:** School of Psychology, Cardiff University, Cardiff, UK; Unit of Clinical Psychology, San Paolo University Hospital, Milan, Italy; Department of Philosophy and Moral Sciences, Ghent University, Gent, Belgium; Department of Public Health and Primary Care, Ghent University, Gent, Belgium

**Keywords:** end of fertility treatment, psychosocial care, IVF/ICSI outcome, wellbeing, psychology

## Abstract

In medically assisted reproduction (MAR) success has mostly been measured in terms of achieving (healthy) livebirths. We argue this focus is too narrow and that success should be measured in terms of alleviating patient suffering caused by an unfulfilled child wish. The major implication is that clinics must better tailored care to effectively support patients who do not have child(ren) with treatment. First, we argue that clinics have a duty of care towards patients for whom MAR does not result in children because this is a common treatment outcome, because treatment is burdensome and creates new losses for patients, and because the field has the necessary expertise to provide support and it is part of patient-centred care. Then, we examine concerns about the adequacy of addressing the possibility that treatment may end without children, namely, that this may hinder patients’ hope and put them off doing treatment, and that it may be perceived as a sign of clinical incompetence, as well as concerns about the required skill set. We end with a set of research-informed recommendations to promote healthy adjustment to ending fertility treatment without children. These focus on the need to reconceptualize ‘success’ and ‘failure’ in MAR, to promote open discussion about the possibility of treatment not resulting in children and encourage patients to develop ‘plan(s) B’, to support patients who end treatment without children, and to create the organizational structures needed to support clinics and healthcare professionals in this endeavour.

## Introduction

Having children is viewed as playing a central role in many people’s lives, from providing meaning to support in older age. For an increasing number of people, parenthood is being achieved through medically assisted reproduction (MAR). Consistently, success in MAR has mostly been measured in terms of achieving (healthy) livebirths. We argue that this focus is too narrow, and that success should be measured in terms of alleviating suffering caused by an unfulfilled child wish (Mertens and Mertes, 2023). The crucial difference is that fertility clinics need to better tailor their care towards effective support for patients who do not have child(ren) with treatment (Gameiro *et al.*, 2013).

## Fertility clinics have a duty of care towards patients who do not have children with treatment, for four key reasons

### First, because not achieving a livebirth is a common outcome of treatment

Many patients who start treatment will end it without children. In the UK, from 107 347 women who started IVF between 1999 and 2007, only 47 189 (44%) had a child after up to eight IVF cycles. The most optimistic estimates indicate that four in each ten patients who do three IVF cycles in the UK or other Western countries will be in this situation (Gnoth *et al.*, 2011; McLernon *et al.*, 2016, 2021; Troude *et al.*, 2016; De Neubourg *et al.*, 2021).

Counselling should therefore prepare patients equally for both outcome scenarios *from the start.* While optimistic counselling portraying a live birth as the expected outcome could seem beneficial as it makes patients worry less about negative outcomes, it is likely to add to the suffering of those who end treatment without a live birth, as this will then be an unexpected event they have not been prepared for. Currently, the likelihood of treatment not working is only implicitly discussed if and when cumulative pregnancy rates are reported to patients, and the emotional burden associated with treatment and its negative outcome(s) are not routinely discussed (van Empel *et al.*, 2010; Rauprich *et al.*, 2011; Leone *et al.*, 2017; Harrison *et al.*, 2022). Ideally, this is replaced by implications counselling, whereby patients are encouraged to reflect about how they feel about potential future scenarios/outcomes, as guidelines routinely recommend for decision-making about different treatment choices (Boivin *et al.*, 2001), e.g. third-party reproduction (van Empel *et al.*, 2010; Rauprich *et al.*, 2011; Leone *et al.*, 2017; Harrison *et al.*, 2022).

### Second, because treatment is psychologically burdensome and creates new losses

Fertility treatment adds to patients’ suffering in different ways. In the treatment process, new embryos are created that many times result in ‘new’ losses over ‘what could have been’, either because they do not lead to a pregnancy, or because they lead to a miscarriage. The emotional burden of treatment has been extensively documented and is mostly associated with the emotional roller coaster of repeatedly building hope despite uncertainty and losing it with news of negative results (Verhaak *et al.*, 2007). Some patients argue that the way treatment is organized intensifies their desire for children and, consequently, their suffering with associated losses (Carson *et al.*, 2021; Tsigdinos, 2022). Meta-analysis shows that indeed, those patients who report a stronger child desire through treatment are at higher risk for maladjustment during and in the aftermath of treatment (Rockliff *et al.*, 2014; Gameiro and Finnigan, 2017). Ending treatment without children triggers intense grief that is associated with moderate to large impairments in mental health and wellbeing (Gameiro and Finnigan, 2017), from which one in ten patients never recover (Gameiro and Finnigan, 2017). To the extent that psychosocial suffering is a product of or intensified by fertility treatment, clinics have a duty to address it, and patients more and more advocate for this (Takhar, 2022).

### Third, because the field has the necessary expertise to support patients

Fertility clinics have expertise about how to support patients for whom treatment does not fulfil their desire for children (e.g. Hammer-Burns, 2004; Boivin *et al.*, 2005; Gameiro and Finnigan, 2017) and it requires minimal efforts to adjust care in the MAR trajectory in a way that equally benefits patients who will achieve a live birth and those who will not. Given the significant benefits for patients and the minimal sacrifices required from clinics, this is an example of a so-called ‘easy rescue’ (Rulli and Millum, 2014) and thus lack of action is unethical.

The literature indicates that nine in ten people eventually adjust to ending treatment without the children they wish for, but patients describe this loss as devastating and the adjustment process as difficult, prolonged (2 years on average), and marked with daily suffering and social isolation (Gameiro and Finnigan, 2017). Clinics should contribute to ease this adjustment process, but research indicates a lack of investment in this endeavour (Frederiksen *et al.*, 2015; Kraaij *et al.*, 2016; Rowbottom *et al.*, 2022; Warne *et al.*, 2023) and patients feel abandoned by their clinics and left to their own devices, expressing frustration and dissatisfaction (Groh and Wagner, 2005; Gameiro and Finnigan, 2017). Tellingly, from 86 evidence-based recommendations presented in the ESHRE Guidelines for Routine Psychosocial Care in Infertility and Assisted Reproduction, only seven (8%) focus on supporting patients for whom treatment does not work (Gameiro *et al.*, 2015). At a societal level, there is low public recognition of the grief associated with ending treatment and there are no mourning rituals (Braverman, 1996). There is some evidence to suggest that the current lack of investment may compound patients’ difficulties by not enabling them to return to the clinic for support (Peddie *et al.*, 2005), not preparing them for the difficult emotions that support may initially trigger (Rowbottom *et al.*, 2022), and not contributing to higher awareness of this topic within primary or mental-health care, healthcare routes patients may also pursue (Gameiro *et al.*, 2016).

### Fourth, because it is part of patient-centred care and what patients desire

Multiple recent primary and evidence-synthesis studies clearly indicate patients are open to and value information about the negative aspects of treatment (Peddie *et al.*, 2004; Dancet *et al.*, 2010; Harrison *et al.*, 2021, 2022; Sousa-Leite *et al.*, 2023). They want to have a realist overview of what their treatment journey will look like before they embark on it, including of the probability of negative outcomes, so that they can better prepare by developing coping skills, anticipating decisions they may have to make, and knowing how to access support when needed (Aarts *et al.*, 2011; Harrison *et al.*, 2021; Sousa-Leite *et al.*, 2023). A very recent survey showed that nine in ten patients reported being willing to discuss the possibility and implications of their treatment not working while they are still undergoing treatment, with seven in ten stating the best time to do so is before doing their first cycle. Patients reported such conversations should focus on providing an overview of their whole treatment pathway and its potential negative outcomes, imparting knowledge and skills to better process loss and sustain a hopeful outlook if treatment ends up not working, and being informed about how to access emotional support and pursue other routes to parenthood and alternative life goals (Sousa-Leite *et al.*, 2023).

## Is it feasible to promote healthy adjustment to ending treatment without children while patients are still undergoing treatment?

In this section, we discuss the most prevalent concerns about the feasibility and adequacy of addressing the possibility of treatment ending without children highlighted in the literature.

### Forewarning that treatment may not work might hinder patients’ hope and put them off undergoing treatment

Healthcare professionals (HCPs) often express concerns about discussing negative treatment outcomes with patients because they do not want to be perceived as unsupportive or discouraging, nor drive patients away from the clinic (Harrison *et al.*, 2022). Similarly, most patients think hope and optimism are important and that too much negativity before treatment starts is not appropriate (Harrison *et al.*, 2022; Sousa-Leite *et al.*, 2022). While patients foregoing treatment based on proper information on all possible outcomes is not problematic, and in fact preferable to patients only pursuing treatment because they are ill-informed, the fear that patients may not pursue treatment because they do not feel supported is a valid concern. However, maintaining a positive and supportive attitude does not equal ignoring the potential of unwanted outcomes.

Indeed, much psychological theorizing and research indicate that people regulate their hope in multiple ways and that just ‘thinking positively’ is not always helpful (Snyder, 2002; Heckhausen *et al.*, 2010). For instance, people who report negative expectations when waiting for news feel more anxious than positive thinkers while waiting, but also feel less dismayed when the news they receive is bad, compared with those who report positive expectations (Sweeny and Shepperd, 2010). Thus, hope can have negative consequences, if it turns out to be ‘false’ hope. Furthermore, some people do not only plan for achieving their desired outcomes but consider a matrix of competing possibilities. Research showed that making plans about how to cope with barriers and blockages to personal goals can contribute to reducing intrusive painful thoughts about such goals, even when no actual action is taken or progress is made (Masicampo and Baumeister, 2011). Overall, the research suggests that fostering hope during treatment is not equally beneficial to everyone and that being hopeful does not equate to ignoring potential negative outcomes. A systematic review also showed that patients only indicate losing faith in treatment or perceiving they have poor prognosis as reasons for having stopped treatment in 5% and 9.5%, respectively, of the times they were presented with these options (whereas, for instance, the physical and psychological burden of treatment is chosen 25% of times; Gameiro *et al.*, 2012).

Despite this evidence, research indicates that as few as two in ten patients report their fertility team acknowledged the possibility of treatment not working (Sousa-Leite *et al.*, 2023). Some patients perceive they are rushed through the IVF process without being fully informed and without much consideration of the negative impact of treatment (Carson *et al.*, 2021; Harrison *et al.*, 2022). One consequence may be that patients are unprepared to cope with negative results, which trigger depressive symptoms, a decline in motivation and a need to rebuild strength and hope (Bailey *et al.*, 2017; Gameiro *et al.*, 2020). As patients themselves argue, putting more emphasis on forewarning can help them to better prepare and take ownership of treatment (Harrison *et al.*, 2022). The challenge that research needs to address is how to talk about possible negative outcomes in a comprehensive but hopeful and sensitive way. Another consequence may be that some patients perceive to be given false hope or even exploited into doing cycles very unlikely to work (Carson *et al.*, 2021; Tsigdinos, 2022).

### Forewarning that treatment may not work may be perceived as incompetence

Qualitative research indicates HCPs perceive an institutional, professional, and personal sense of failure when fertility treatment does not result in children (Leone *et al.*, 2017). Their narratives indicate they move from a position of omnipotence, given their ability to offer the miracle of generating life, to a position of impotence, when faced with repeated cycles that do not result in pregnancy (Leone *et al.*, 2017; Fedele *et al.*, 2020). In conjunction with high patient expectations, these conflicting emotions can result in performance anxiety and avoidance of decision-making around ending treatment (Fedele *et al.*, 2020). In this context, HCPs may feel their role is to keep patients hopeful until treatment eventually succeeds, potentially leading patients to do more cycles than initially anticipated. HCPs may also find it difficult to temper patients’ expectations, even when these are perceived as unrealistic, and especially when patients are extremely committed to pursue their parenthood goals (Grill, 2015; Klitzman, 2016; Leone *et al.*, 2017).

More transparency and discussion of average success rates in the field of MAR prior to the start of treatment may contribute to disentangling the notion of competence from the outcome of treatment cycles. This approach can also facilitate patients’ decision-making process about cycle uptake, unburdening HCPs from the moral dilemma of deciding for whom and when the option of stopping treatment should be introduced (Grill, 2015; Klitzman, 2016). An interview-based study with HCPs showed that when they perceived that the decision to end treatment was discussed, shared, and accepted by patients, patients trusted them more, and this increased their sense of professional fulfilment (Leone, 2023). In sum, the way that end of treatment is communicated with patients can shape trust and quality of care.

### HCPs feel ill-prepared and lacking appropriate skills to engage in conversations about treatment ending without children

Conversations about negative treatment outcomes are hard for patients and staff alike. Both fear these may trigger anxiety in patients, which has been confirmed by research (Devroe *et al.*, 2022), and a minority feel it makes no sense to discuss something that may not happen (Harrison *et al.*, 2021; Sousa-Leite *et al.*, 2023). Qualitative evidence indicates HCPs find it hard to use personal discretion in deciding with whom and when to have end-of-treatment conversations and feel unprepared to introduce such sensitive topics and manage the difficult emotions these may trigger (especially anger, but also sadness, disappointment, frustration) (Simpson and Bor, 2001; Grill, 2015; Leone *et al.*, 2017). HCPs report feeling conflicted in their own decision-making about discussing end of treatment with patients because they lack explicit criteria, policies, or a formal ethical framework to guide such decisions (Klitzman, 2016; Leone, 2023).

However, HCPs cannot escape these conversations and have the duty not to avoid these nor to add further suffering to patients by not investing in how to approach these well. Indeed, patients list insensitive communication from HCPS as bad news in itself and compounding any negative impact of bad fertility news shared (Gameiro *et al.*, 2024). A call to action for more research and professional development opportunities is required here, as it is unquestionable that more communication training is needed to support HCPs in approaching what is one of the hardest tasks in their jobs. Psychologists and counsellors will have this expertise and can lead knowledge transfer within multi-disciplinary teams, but only a few evaluated initiatives can be reported so far (e.g. Garcia *et al.*, 2013; ESHRE, 2024).

## Recommendations to promote healthy adjustment to ending fertility treatment without children

In this section, we offer research-informed recommendations to support clinics interested in promoting patients’ adjustment to ending treatment without children, which are summarized in Table 1.

**Table 1.** Summary of research-informed recommendations to promote fertility patients’ adjustment to ending treatment without children and tips and resources to support their implementation.

Reframe fertility treatment success and failure	Tips and resources
Use descriptive language to talk about the outcome of fertility treatment, rather than value-laden language Focus more on the psychosocial outcome of treatment than on the physical outcome (i.e. healthy livebirth rate) when measuring treatment success Do not frame cycle(s) that do not result in pregnancy or childbirth as *failed* Do not refer to not doing (more) cycles of treatment as *dropout, non-adherence, non-compliance, giving up*, or *dropping out*	Say—*The cycle did not result in a pregnancy/livebirth*; *Treatment ended without children; The cycle did not work as expected* instead of*—The cycle failed/It was a failed cycle.* *Say—Patients sometimes decide not to do more cycles of treatment/end their treatment/pursue other goals* instead of—*Patients sometimes give up/dropout/discontinue treatment* (Harrison *et al.*, 2021, 2022, 2023)

**Promote open discussion about the possibility of treatment not resulting in children**	

Provide repeated and transparent information about cumulative livebirth rates in fertility treatment Complement this medical information with psychoeducation about the implications of all possible outcomes of treatment Encourage patients to develop a treatment plan before they start any treatment cycle Promote shared decision-making about doing (more) cycles or not	Visit www.myjourney.pt/clinics for guidance on how to initiate conversations with your patients about the possibility of treatment not working, 10 facts about ending treatment without children you should tell your patients, common questions patients ask and suggestions on how to address these, and resources you can share with your patients. For detailed information of what patients expect from these discussions, see Sousa-Leite *et al.* (2023). Signpost patients to www.myjourney.pt/patients if they prefer to explore this possibility on their own. See (Harrison *et al.*, 2021, 2022, 2023) for further information on treatment planning.

**Encourage patients to develop and discuss ‘plan B(s)’**	

Encourage patients to remain engaged with other valued life goals while they are undergoing fertility treatment Encourage patients to develop ‘plan B(s)’ they can fall back upon in case treatment does not result in children Systematically reassure patients that not doing (more) cycles of treatment can be a sensible decision to make	Say—*Because treatment can be very energy-sapping, you should definitely pay attention to how you top up your energy levels. This could be engagement in hobbies, developing sporting activities, looking for experiences in nature, going to concerts, practising meditation, etc. Patients who continue engaged with their hobbies or other valued lifegoals tend to feel better during their treatment journey.* Say—*Although we all want and are working together to maximize your chances of having a child, it is possible this may not happen. Deciding now on ‘plan B’ to pursue in case treatment does not work as expected can help you face this unknown. You can think about individual or shared [when patients are a couple] plan B(s) that you think may bring you pleasure, meaning, and fulfilment. You can write down your ‘Plan B’ now and put it in a safe place where you know you can find it later, if you need it.*

**Support patients who end treatment without children**	

Implement an end-of-treatment consultation to explain why treatment did not work and to provide information about other routes for parenthood or life without children, as per patients’ preferences Remind patients about what most people experience when treatment does not work and promote reflection about what can make this transition easier for them Inform patients of and signpost to available support resources Refer patients at risk for maladjustment to specialized psychosocial care (infertility counselling or psychotherapy)	Signpost patients to My Journey (www.myjourney.pt), a multilingual webapp designed to ease acceptance of an unfulfilled wish for children that clinics can freely offer to their patients. Its evaluation showed that people who use it experience clinically significant improvements in their wellbeing within 10 weeks, manifested as feelings of joy, contentment, and fulfilment, as well as improvements in their mental health within 6 months (Rowbottom *et al.*, 2022)

**Create organizational structures to support clinics and HCPs**	

Educate about the patient experience of treatment Provide evidence-based training on how to share bad news with patients Promote self-care skills for staff Create opportunities for multi-disciplinary team debriefs about difficult patients encounters Promote discussion and team-based decision-making about end-of-treatment care provision	www.myjourney.pt/clinics offers information about the experiences and concerns of patients who end treatment without children Gameiro and Finnigan (2017) provides a comprehensive synthesis of the patients’ adjustment in the aftermath of treatment Baile *et al.* (2000) provides an evidence-based approach to sharing bad news with patients that can be tailored to fertility care

### Reframe fertility treatment ‘success’ and ‘failure’

We need to reflect on why we only consider the birth of a (healthy) baby as success in MAR. Unless one adopts an explicitly pronatalist ideology, this is not because producing children is morally laudable, but rather because by helping people conceive, they are helped in achieving goals *they* judge central to their wellbeing and purpose in life. The association between parenthood, wellbeing, and happiness is complex (see Mertes, 2017; Mertens and Mertes, 2023) and patients may be mistaken in their expectation that becoming a parent will substantially increase their wellbeing (Wischmann and Thorn, 2021). For the purpose of this article, it suffices to say that an unfulfilled wish for (more) children can cause intense suffering and is more strongly associated with wellbeing outcomes than parental status (Gameiro *et al.*, 2014). The logical conclusion is that alleviating this suffering should be the main underlying rationale for fertility treatment and underpin the definition of successful treatment. We therefore propose a new definition of treatment success in infertility care. Fertility treatment is successful when the suffering that accompanies subfertility and infertility is alleviated throughout and beyond the treatment trajectory. Successful treatment can either end with a healthy live birth, or with a state in which patients succeed in coping with their infertility in such a way that it no longer has a detrimental effect on their well-being.

Reframing success of MAR in this way has consequences. First, the language used within the field needs to be scrutinized. While it is true that ending treatment with a healthy child is the preferred outcome or ‘plan A’ for patients, we should be careful about framing *not* achieving this ‘plan A’ in terms of failure, because there are other options (plan B) that can be explored to reach the goal of relieving patient suffering. We must also avoid framing pursuing other avenues as non-compliance, non-adherence, abandonment of treatment, giving up, or dropping out (Lee, 2022; Mertens and Mertes, 2023), which have very negative and culpable connotations. Besides misplaced feelings of guilt and incompetence in patients and HCPs when treatment does not have the desired outcome (Leone *et al.*, 2017; Fedele *et al.*, 2020; Carson *et al.*, 2021), such language may contribute to HCPs and patients continuing treatment for longer than they had initially planned or judged desirable (Daniluk, 2001a). Although changing language is never easy, it is important that we reinvent the way we talk about the end of treatment. Suggestions are to simply be descriptive and refer to ‘ending treatment with or without children’, ‘treatment (not) resulting in a pregnancy or livebirth(s)’, as per language used in this article.

Second, HCPs need to weight the suffering caused by an unfulfilled wish for children against the suffering caused by treatment. Given how uncertain the treatment outcome is, the wellbeing of patients during and after treatment (regardless of its outcome) should be a major concern driving clinical decision-making. Patients can be willing to bear the treatment burden but only if they gain effectiveness (von Estorff *et al.*, 2024), which becomes less likely as patients progress through consecutive cycles. Firstly, clinics can minimize the risk for treatment to become an unstoppable rollercoaster by encouraging patients to proactively consider the aspects of treatment they can influence even before they start it, e.g. how long and how much do they want to spend doing treatment and how many cycles they are willing to do (Van den Broeck *et al.*, 2010; Wischmann and Thorn, 2021; Harrison *et al.*, 2022). Secondly, clinics can promote active and shared decision-making about doing (more) cycles of treatment, rather than both parties assuming that treatment continuation is the default option (Klitzman, 2016).

Overall, patients and clinicians are expected to benefit from the message that they do not have to go to extremes for their efforts to be valued, and that stopping treatment ‘in time’ (and moving towards a plan B) can be a better decision for patient wellbeing and good clinical practice than desperately holding on to a small chance of plan A succeeding.

### Promote open discussion about the possibility of treatment not resulting in children

A first step in preparing patients for the possibility of treatment not resulting in children is to inform them repeatedly and in a transparent manner about the expected chance of achieving a livebirth. People *not* achieving parenthood after IVF treatment remain largely invisible and unvalued in the public domain (De Lacey, 2002), making it difficult for those going through treatment to identify with this group, rather than with the group who have children, and fertility patients refer to being socialized to expect a happy ending (Daniluk, 2001a). Waiting rooms of fertility clinics are oftentimes decorated with birth announcements and baby pictures on a ‘wall of hope’, which can create the false impression that those in the room who did not yet achieve a pregnancy are the exception, rather than the rule. Moreover, even patients who have been counselled about their chances of achieving a live birth continue to overestimate them, believing that they are able to ‘beat the odds’ (Miron-Shatz *et al.*, 2021).

While conversations about livebirth rates and the high probability of an embryo transfer not resulting in a pregnancy are difficult for HCPs and patients alike, they help in managing patients’ expectations, especially for patients with poor prognosis (Devroe *et al.*, 2022). Patients are reported to appreciate honest and realistic information about what they can expect without sugar coating, even when that information is not always what they would have liked to hear (Daniluk, 2001a; Borghi *et al.*, 2019), and feel duped by practitioners offering them ‘false hope’ throughout their treatment (Daniluk, 2001a). To support adaptive coping in case of the treatment not resulting in children, medical information should be complemented with psychoeducation about what a negative outcome means for most patients.

### Encourage patients to develop and discuss ‘plan(s) B’

Clinics and HCPs need to have a consistent treatment narrative. They should not explicitly or implicitly communicate that having genetically related children is the only successful outcome, and then replace this message after a couple of failed cycles by one entailing that donor conception is of equal value to genetic parenthood, only to then, after that option fails, tell patients they can be happy without children.

Instead, we propose a new treatment narrative in which hope is framed in multiple ways and consistently communicated to patients from the very start. Such narrative should convey that:

- the clinic will provide patients with the best treatment (and care) possible to help them have the children they wish for.
- happiness and fulfilment are possible even when treatment does not result in children as, with time, most people recover from grief, reporting a sense of survival and personal and spiritual growth, as well as greater ability to value what life has to offer (Daniluk, 2001b; Gameiro and Finnigan, 2017).
- ending medical treatment is not the same as end of care, and clinics will support patients in coping and navigating through the implications of either treatment outcome (Gameiro *et al.*, 2013).

Adopting this narrative has implications for care provision. Firstly, HCPs can advise patients to remain engaged with other lifegoals that already give them pleasure and fulfilment. Secondly, HCPs can support patients in working out their ‘plan B(s)’ at an early stage of treatment (Wischmann and Thorn, 2021). Systematic review shows that keeping parallel goals to having children is associated with higher wellbeing during and in the aftermath of treatment (da Silva *et al.*, 2016). Thirdly, HCPs can ensure that ‘plan B(s)’ remain in sight and are discussed at key moments in treatment (Van den Broeck *et al.*, 2010), for instance, at the start of treatment or after each failed cycle or, at the very least, after three or all funded cycles were attempted. In this context it is important that HCPs send the message that stopping treatment is always an option and can be a positive and brave decision to make, while making sure to reassure patients they did ‘enough’. HCPs also need to keep in mind that a ‘plan B’ is not necessarily donor conception or another medical intervention but can be located outside the clinic.

In sum, we advise HCPs to systematically build in moments for patients to consider all possible treatment outcomes and to actively reflect on what is best for them moving forward. Currently, there is a discrepancy between the number of patients who report having properly discussed end of treatment with their HCPs and the number of patients who end treatment without a baby, meaning that many patients who needed and could have benefited from this conversation did not receive it (Sousa-Leite *et al.*, 2023). Even though some patients may not have the mental room to engage in these conversations when HCPs want to approach them, it is necessary that HCPs systematically signal they are available to have this conversation whenever patients are ready. It is the clinician’s duty to extend the invitation to the patient, and the patient’s right to either embrace or reject this invitation.

### Support patients who end treatment without children

After a decision to stop treatment has been reached, it is important to support patients in the rough patch they have ahead of them. Patients have reported feeling abandoned by their fertility team when they decided to discontinue treatment or when no more treatment options were available to them (Gameiro *et al.*, 2013; Gameiro and Finnigan, 2017). If the success of fertility care is defined in terms of the wellbeing of patients (see above), then care cannot stop when the last cycle ends. While clinicians may feel less competent to guide patients through this phase of the care pathway, an end-of-treatment consultation plays a crucial role in helping patients understand why treatment did not work and reach closure (Groh and Wagner, 2005). Even if clinicians cannot pinpoint exactly what did not work, they can remind patients of the limitations of MAR, as this helps patients accept their situation (Gameiro and Finnigan, 2017). At this last appointment, HCPs also need to acknowledge patients’ efforts to have children and reassure them they explored ‘enough’ options (Gameiro and Finnigan, 2017). For patients whose ‘plan B’ includes exploring alternative routes to parenthood, information about these should be offered. Finally, HCPs need to inform or remind patients of what they can expect moving forward and reassure them they can access support immediately or later, when needed or desired.

### Create organizational structures to support clinics and HCPs

Clinics and their staff should be supported in coping with the demands and burden of discussing negative treatment outcomes. First, communication skills training about how to share bad news is highly relevant to enable staff to confidently engage with these conversations. The ability to communicate well is a professional competency of any HCP and not just a personal aptitude. Clinics should allocate resources and protect time to allow staff to participate in evidence-based communication training, which has been proven to improve confidence and actual performance (Johnson and Panagioti, 2018), with the use of specific protocols being associated with lower perceived stress when communicating with patients (Simpson and Bor, 2001). Second, HCPs need to be supported in addressing not only intense patient emotional reactions but also their own emotions, such as guilt and a sense of failure. This is relevant for their own wellbeing and to prevent such emotions from interfering in their clinical practice (Leone *et al.*, 2018; Facchin *et al.*, 2020). Within a multidisciplinary team, mental health professional (MHPs) can be involved in training and supporting other team members (Gameiro *et al.*, 2013; Grill, 2015; Sax and Lawson, 2022), for instance by imparting communication and self-care skills, creating opportunities for debriefs after difficult patient encounters, or educating about the patient experience of treatment. Specific to this manuscript’s topic, MHPs can support other staff in discussing critical situations about ending treatment, e.g. when there is a disagreement between patients and staff and when moral and ethical dilemmas arise (Grill, 2015; Klitzman, 2016), so that clinical decisions are shared within the team and lighten the burden of personal responsibility (Leone, 2023). Finally, the field should be investing in growing the evidence base that informs provision of end-of-treatment care and developing tools to aid in this endeavour (e.g. development and evaluation of support interventions, shared decision-making aids) so that HCPs feel reassured they are following best practice evidence-based recommendations.

## Conclusion

The high likelihood of treatment not resulting in a newborn creates an ethical imperative for clinics to prepare and support patients through this potential outcome. This article offers several research-informed recommendations to support clinics in this endeavour. Regulatory bodies need to monitor provision of end-of-treatment care to ensure all fertility patients receive high-quality care.

## Data Availability

No data are associated with this manuscript.
